# Patents in Any of the Different Branches of Medicine

**Published:** 1852-07

**Authors:** W. R. Handy


### ARTICLE XI.

### *Patents in any of the Different Branches of Medicine.* By W. R. Handy, M. D.

The subject of the *propriety* of patenting any *improvement, discovery* or *invention,* which bears upon the art of medicine in any of its several branches, is now engaging the attention of the medical and dental professions, and calling forth the opinions of some of their members on this point.

It is a subject of great interest—involving, as it does, that highest of all temporal interests, viz. the preservation of human life—as well as bearing directly upon the noble art of medicine, touching its honor, dignity, and world-wide benevolence.

It is a subject admitted to be of some difficulty, and about which, honest and intelligent minds have differed, and which, therefore, makes it the more necessary, that some general agreement of sentiment, as well as harmony of action, should universally prevail.

To this end, we purpose making simply a few brief remarks on the point at issue.

The law recognizes the right of an individual to any invention, improvement or discovery in the construction of machinery of any kind, and protects such right by granting a patent, so as to remunerate him for the labor, time and money he may have expended in perfecting such machinery designed to add to the bodily comfort of man.

*Copy-rights* are also secured to authors for their loss of time, labor and money, in adding to the intellectual improvement of man ; and the general voice of the people seems to be, that such laws are right and just, as no individual can be expected to make such sacrifices without remuneration.

But, when you come to the subject of disease—the saving of human life from suffering and death—the question then of protective right to improvements, discoveries and inventions, does not, by any means, meet with the same unqualified approval.

Why, we ask, this difference ? And what the origin of such feeling ?

It may be said, that if the steam engine wafts us with rapidity from point to point, either for pleasure or business, and the electric telegraph conveys, with lightning's speed, a world of thought, sayings and doings, in, almost, an instant of time, to the remotest places ; and the cotton gin and loom, with all their curious and complicated appliances, furnish suitable material for the clothing of the body ; and the stereotyped volumes of men of genius for informing the mind. If all such productions as these, it is asked, merit, and justly claim, the protection of the law, how comes it that improvements, discoveries and inventions, relating to the far higher interests of preserving life, should be considered unworthy of a like protection ?

It is true that patents in medicine have been obtained, but these are comparatively few in number, and even these few, we are of the opinion, are very far from receiving the approval of the great body of the profession, or even of the people at large.

Why, it is again asked, is it, that discoveries and inventions relating to disease, should not be patented ? It has been already stated, there was no legal difficulty in the way ; but, only the general feeling, both professional and unprofessional, which was against it, and it is just this feeling which seems to form the reason, why the opinion is so prevalent that patents, in all such cases, are wrong.

Now, if such feeling as this were only occasional, and occurred but in few cases, it might be thought of little value, and scarcely worthy of notice; but when, on the contrary, such feeling becomes universal; when it is found in the savage as well as the civilized man; in the ignorant as well as the informed, the inference seems fairly to force itself, that such feeling is fundamental to our nature, of binding authority, and enforcing a moral obligation, which every one feels as instinctive to his very being.

Now, if this be true, the common opinion of mankind is against all patenting whatever in medicine, and regards all such privileges as so many violations of the moral rights of the people, as manifested through the moral sense, in determining what is right and what is wrong.

Such a view as this, it would seem, places the medical profession upon a great moral basis, and makes the every day business of that profession, viz. healing the sick, of such a high and sacred character, and of involving such weighty responsibilities, as not, for a moment, to be put in contrast with, or placed by the side of simply dollars and cents, when the life of a human being is at stake; and he, therefore, who would obtain an exclusive privilege, by patent, to save men's lives, and that only such as would choose to pay for, and obtain the healing virtues of such patent, must be doomed to die, would at once be pronounced a very monster of avarice and cruelty, a being in human shape, but thoroughly destitute of all the feelings of a common humanity, as well as of common decency.

In a word, the *art of healing* has more than once been styled a divine art—one not to be acted out, and measured by pecuniary profit—but one to be as free as the air we breathe, and one consequently allowing no restrictions or exclusive privileges, when designed by the God of nature as the common heritage of all mankind.

Hence, the medical profession, with the noble magnanimity of a liberal science, have always discountenanced every thing like nostrums, and all secret remedies for the cure of disease, and further, to refuse fellowship with the man who will thus, either knavishly or ignorantly, trifle with the life of his fellow man.

The medical profession is regarded as a common profession —as one great brotherhood for the common good—consequently, having one common interest, and thus, by the common contract, necessarily having a common share to the accumulative property belonging to the common stock.

By such an agreement, no one member, therefore, can claim any exclusive right or privilege over that of any other member, and he who would set up such claim, by taking out a patent, thus virtually nullifies his allegiance to the compact, severs his connection with the profession, and is no longer regarded as an honorable member; and why? Simply for laying claim to that which does not belong to him. It is the property of a common profession, consequently belongs to all, and no one, hence, can have an exclusive right.

What each one can add to the common stock, it is considered, not only that he receives, out of that common stock in return, a full equivalent, but further, a measure fully heaped up, and running over; and that mind, it is believed, must be possessed of no small share of vanity, to think that all other minds have contributed nothing to the common stock, and that from this universal poverty of mental contribution, this alone luminary, receiving nothing in return, modestly comes out and claims a patent for extra contributions of services rendered.

With this explanation of the principle, involving the right of patenting in general medicine, we come now to consider whether this same principle is applicable, or not, to all of its specialties.

If the principle be applicable to the whole, it most assuredly must be so to the several parts, as, out of these same parts the whole is made; and, though it may not apply with the same force to each, yet all must feel its influence in a greater or less degree. And it is because the working of this principle seems so faint when applied to what are called the mechanical branches of medicine, that some very honest minds have been led to the conclusion, that the principle does not apply at all, and that patents, therefore, are justifiable upon the same grounds, as all other improvements of the day. As, for instance, in surgery and dentistry, where various kinds of contrivances, such as *splints*, *trusses*, instruments, &c., claim the right of being patented, without it is thought violating that general law relating to the improvements necessary to the successful treating of disease. In other words, the patient will get well without my improvement; therefore, I have a right to a patent; but, as he can get well much sooner with my improvement or discovery, therefore I have full right to make all patients suffer who will not buy my secret, as well as cheat my professional brethren by not rendering to them as they have rendered to me.

Now, in the mechanical application of splints to a broken leg, the great principle for a successful cure is, that the leg shall be kept at rest—the splints do this—and consequently aid in the successful cure of the limb ; and, therefore, we think, has no more right to a patent, than the medicine employed for subduing inflammation, fever, &c., which is also equally necessary for a successful cure.

But, if it be said the limb would get well without the splints, all good surgery denies the fact, and only admits such cases, when they occur, to be rare exceptions to the general rule, of their indispensable necessity.

And even admitting the bone would unite without the splints, yet, who would be willing, under the shelter of a patent, to inflict all the suffering which, as a sound pathologist, he knows must necessarily follow without their use. In other words, the splints must be regarded as an essential part of the remedial agencies, necessary for the most successful, safe, and speedy cure of the disease.

And so in all surgical appliances, we hold, the principle is equally binding; as they all have a direct bearing upon, and especially designed to aid in the cure of disease; and though their aid may be considerd humble in the part they take, when the propriety of a patent is questioned ; but of vast importance when it is to be obtained—yet, as it is allowed to be an aid, it matters little whether that aid be small or great—it forms an indispensable link in the great chain of cure, and should not therefore be withheld by a patent.

In dentistry, the patenting of inventions is no uncommon occurrence, and there does seem, at first view, to be some greater plausibility of the propriety of an exclusive right in this profession, than in that of general surgery; but if the general principle contended for be correct, the dentist has no better claim to a patent than the physician or surgeon.

It is true, at one period of the world, dentistry was not regarded as a science; nor acknowledged as a legitimate specialty of medicine; the teeth were not regarded as organs; but rather viewed as so many foreign bodies placed in the mouth, after the manner that nails are driven into a board.

*Gomphosis* was the term used to express the kind of articulation that was supposed to exist between the teeth and mouth; that is, that like the nails of the board, there was little or no vital union, such as we find exists between organ and organ; and that, consequently, all operations upon such bodies, could, in no way, sympathetically affect either adjacent organs or the system at large. With such views, it is not to be wondered, that mechanical dentistry reigned supreme, and that mechanical dentists should apply for patents. But, now that it is well known, that dentistry is an essential part of medicine, and that the teeth are organised bodies, possessing all the elements of organization, as, viz. in having blood from a common circulation, and nerves from a common sensation, and thus establishing a common sympathy with the general family of organs, thereby make the teeth as the rest of the organs to be endowed with life; and as such, subject to disease, and thus render all the appliances necessary to the cure of their diseases, as not coming under the head of justifiable patents, more than those of general medicine, which have just been considered.

But it is further suggested, that if the individual has retired from the profession, and no longer reaps any of its emoluments, that then he may fairly claim the right of a patent, and the exclusive enjoyment of all the benefits flowing from such right. We reply, that such retirement should not justify the nullification of a solemn compact, and the trampling under foot a moral obligation, to the great damage of suffering humanity—an immense loss to a vast many, and but trifling gain to a very few.

### SELECTED ARTICLES.

